# A Circular

**Published:** 1855-07

**Authors:** 


A CIRCULAR.

A very curious document of this kind fell under our notice a short time since, by sheer accident. The only feature in it which would occasion anything else than a smile with us, is, that it boldly avows that "*the standard of Medical Literature in the Western States is either in reality, or in public opinion, below the standard of that which it mainiains in the East?*"—Now, which of these positions is true? Is the standard really lower, or is it an error of public opinion? One or the other is presumed by the author of this proclamation, and if *either*, he is on one horn of the dilemma. Either the profession of the West is assailed and insulted, or the people are grossly ignorant, for, in a most bombastic style, it proposes to cure the evil, although he has not *diagnosed* it. It exists somewhere, according to the logic used, and it is fair to conclude that the profession is meant, and if so, we maintain if the mass of the medical profession is referred to, it is false—a slander upon the profession of the West, and as one of the sentinels of that profession, we hereby defend them from the charge. Take the practitioners as they are scattered through the country, in the field of active practice, and they will be found to be as well cultivated, as well educated, and as efficient a corps of medical men as is now in the Eastern States, in proportion to the numbers. If the *people* are meant, then *their* intelligence must be below par, which is again without foundation.—There is no proposition to cure this difficulty in the people. It only proposes to remedy the great evil in medical men, by instructing *them*—a scheme which will prove about as successful as a former effort of a similar character some two or three years since. We suppose many of our readers have already received this precious *morceau*, (the real authorship of which there is but little difficulty in determining,) and have already enjoyed a laugh over it.

We shall refer to this again at some future day, when we will examine it more carefully for the satisfaction of those of our readers who have not seen it. One thing is certain that it is a poor compliment to our medical men, after the many encomiums bestowed upon the Western men by the profession of the East, and we have not estimated their spirit rightly if they will much thank the author for this want of respect for their qualifications and standing.

